# Multicenter evaluation of the QuickMIC^®^ rapid AST system in clinical practice: impact on turnaround time compared to routine AST systems

**DOI:** 10.1007/s10096-025-05298-z

**Published:** 2025-10-13

**Authors:** Anna Olsson, Alexandra Rafeletou, Amanda Åman, Nicolette Athanasiou, Celia García-Rivera, Derek Gerstbrein, Brian Mesich, Logan Patterson, Julia Vahturova, Nicole VanZeeland, Andrea Ricart-Silvestre, Matthew L. Faron, Jessica Hoff, Kevin Alby, Marina Ivanova, Juan Carlos Rodríguez Díaz, Cecilia Johansson, Christer Malmberg

**Affiliations:** 1 Gradientech AB, Dag Hammarskjölds väg 36B, Uppsala, 75237 Sweden; 2 Department of Pathology, Medical College of Wisconsin, Milwaukee, WI USA; 3 Alicante Institute for Health and Biomedical Research (ISABIAL), Dr. Balmis University General Hospital, Alicante, Spain; 4 Department of Pathology and Laboratory Medicine, Medical University of South Carolina, Charleston, SC USA; 5 Clinical Microbiology Laboratory, East-Tallinn Central Hospital, Tallin, Estonia; 6 Department of Pathology and Laboratory Medicine, University of North Carolina School of Medicine, Chapel Hill, NC USA; 7 Department of Medical Sciences, Uppsala University, Uppsala, Sweden

**Keywords:** Rapid AST, Sepsis, Antimicrobial resistance, Diagnostics

## Abstract

Increased antibiotic resistance highlights the need for new, rapid antibiotic susceptibility tests to guide therapy, especially in critical disease such as bloodstream infections and sepsis. This study investigates a new ultra-rapid AST system in multiple clinical laboratories, with respect to accuracy, speed and turnaround time in comparison to commonly used AST systems. The QuickMIC system is compared to the commonly used automated AST systems BD Phoenix™ (BD, USA), MicroScan WalkAway plus (Beckman Coulter, USA) and VITEK^®^ 2 (bioMérieux, France) by concurrent testing of incoming positive blood-cultures with Gram-negative bacteria in four clinical laboratories located in the EU and USA, on the basis of agreement of results, time-to-result (TTR, analysis time) and turnaround time (TAT, time from blood culture positivity or blood culture processing to actionable result). A total of 155 patient samples were included, totaling 10 species of Gram-negative bacteria. The overall EA and CA between QuickMIC^®^ GN and each routine AST system was > 95%, while overall bias was within the acceptable range of ± 30%. QuickMIC time-to-result was on average 3 h and 4 min, compared to 9–19 h for the routine systems. The average QuickMIC system turnaround-time ranged from 10 to 11 h 30 min, compared to 22–45 h for the routine systems. The QuickMIC system represents a promising rapid AST technology with potential to reduce time-to-result and overall turnaround-time of clinically actionable AST results compared with the most common routinely used automated AST methods, while maintaining good accuracy and quality of results.

**Supplementary Information:**

The online version contains supplementary material available at 10.1007/s10096-025-05298-z.

## Introduction

The rapid emergence of antibiotic resistance is a threat to modern medicine, and with it the likelihood of successful empirical treatment is decreasing, which is especially concerning in critical infections such as bloodstream infection and sepsis [1]. The incidence of bloodstream infections varies globally, but recent data suggests an incidence of 677.5 cases per 100,000 in 2017, with very high associated costs to society [2]. Receiving suitable antibiotic treatment within 12 h from blood sample collection [3] or 1–3 h after signs of septic chock [4] has been shown to be associated with a reduced mortality, which highlights the need for more precise and rapid diagnostics as antibiotic resistance increases and the likelihood of coverage by initial empirical therapy decreases. Even so, antibiotic susceptibility testing (AST) systems commonly used in clinical routine laboratories worldwide such as BD Phoenix™ (BD, USA), MicroScan WalkAway plus (Beckman Coulter, USA) and VITEK^®^ 2 (bioMérieux, France) reports an actionable AST result (Minimal Inhibitory Concentration, MIC) within 4–18 h, often closer to the later [5]. The current diagnostic workflow and the limited opening hours of most clinical routine laboratories are other reasons for why the time to an actionable MIC often is delayed by multiple days. In addition, late communication of laboratory results to the ward and lack of on-site senior physicians off-hours further limits the time during which the empirical treatment can be adjusted. As a result, having access to more rapid AST results and the possibility of performing same-shift diagnostics and guidance of antibiotic therapy is potentially game-changing for the treatment of critically ill patients.

During the past decade multiple rapid AST systems, usually with an average analysis time of 5–7 h, have been developed: Alfred 60/AST (Alifax^®^, Italy), ASTar system (Q-linea, Sweden), Pheno^®^ system (Accelerate Diagnostics, USA), QMAC-dRAST™ (QuantaMatrix, South Korea) and VITEK^®^ REVEAL™ (bioMérieux, France) [6, 7]. Furthermore, the disk diffusion based EUCAST RAST protocol can deliver AST results as early as within 4 h, but only for a subset of species and antibiotic agents [8–11]. EUCAST RAST has proven to be a valuable tool at a low cost, but it does not provide quantitative MIC values and is more laborious with repeated manual reading (although automation is possible), and the plates can be difficult to interpret at times [12, 13]. For same-shift diagnostics to be fully implemented in hospitals, without the need for 24/7-staffing and highly trained microbiologists, the time to result (TTR) needs to be low enough for the test result to be available before the regular shift has ended. Today, identification of the bacterial species using MALDI-TOF-MS (Bruker, Germany) or rapid molecular detection of the species and resistant genotypes are generally very fast procedures and are useful for guiding treatment in many instances (e.g. infections with Methicillin Resistant Staphylococcus aureus and carbapenem-resistant Enterobacterales, particularly producers of common carbapenemase classes often present on multiplex panels such as KPC, NDM, VIM, IMP and OXA-48) [14, 15]. Molecular methods are however not comprehensive enough to cover the large variability of Gram-negative acquired resistance genes. Therefore, same-shift AST of Gram-negative bacteria using phenotypic ultra-rapid AST with an analysis time ≤ 4 h may have a large clinical value.

**Fig. 1:**
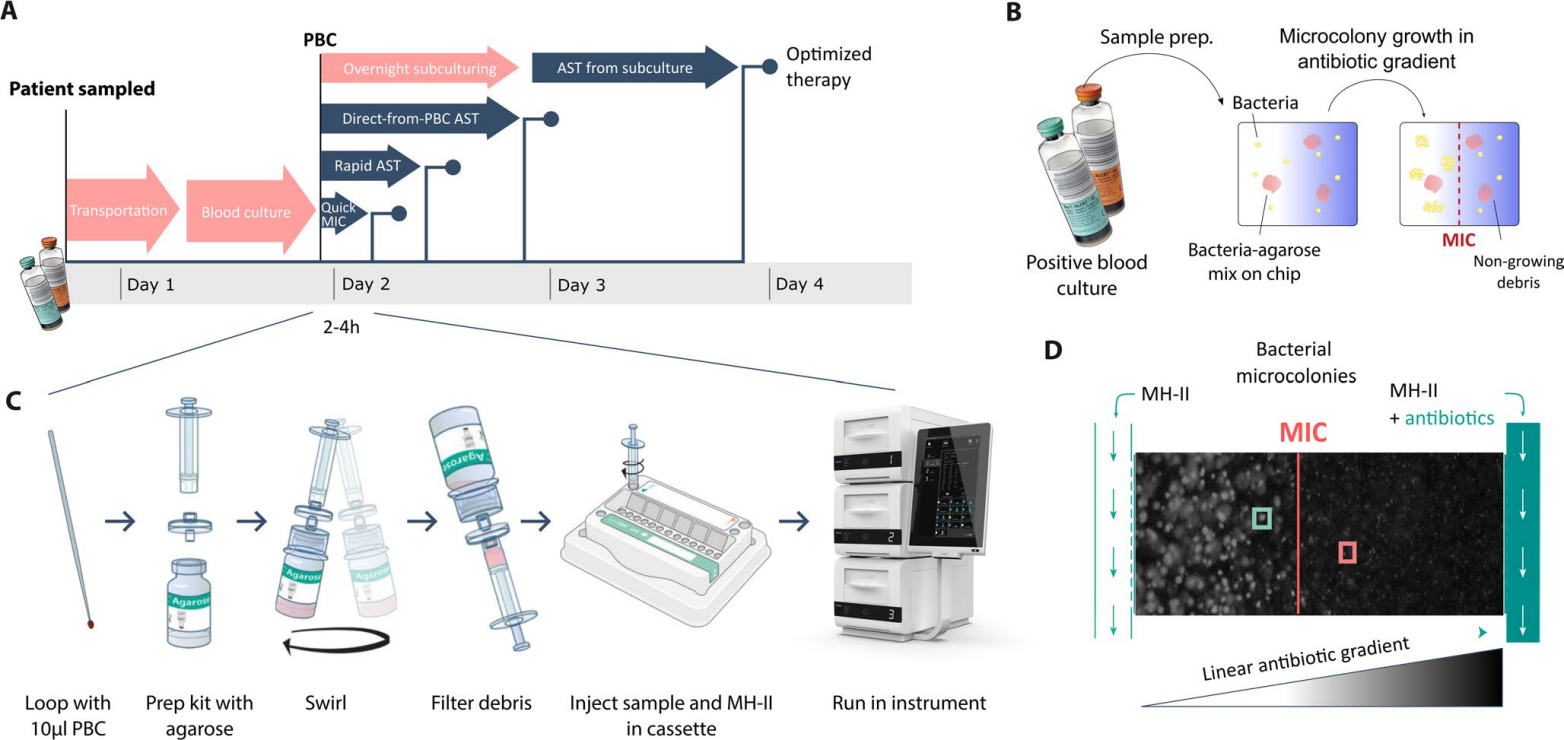
Brief overview of the QuickMIC ultra-rapid AST system and its components. **A**, Overview of QuickMIC workflow in comparison to AST using traditional methods from overnight subculture, traditional methods run direct-from-PBC, as well as typical rapid AST methods with ~ 5–7 h runtime. **B**, Explanation of the QuickMIC measurement process, where sample preparation is performed directly-from-PBC, and bacterial growth on-chip can be discerned from debris by growth. **C**, QuickMIC^®^ GN cassette workflow in detail, where a PBC sample is introduced into the agarose vial, and debris is filtered after mixing followed by injection into the cassette which contains 12 pre-loaded antibiotics. **D**, Detailed explanation of the linear antibiotic gradient formation in each of the 12 test chambers, allowing the determination of a precise, linear MIC

The QuickMIC system (Gradientech AB, Sweden) is a phenotypic ultra-rapid AST system that provides growth-based susceptibility profiles within 2–4 h (Fig. 1) [16, 17]. The operational principle of the QuickMIC^®^ GN cassette is a microfluidics-generated antibiotic gradient through a 3D agarose hydrogel with embedded bacteria, which in combination with solid phase cytometry very sensitively provides the MIC value for 12 different antibiotics simultaneously. This short time to results makes the QuickMIC system a promising candidate for blood culture diagnostics and successful implementation in clinical routine labs without the need for extensive workflow adjustments. The performance of QuickMIC^®^ GN has been evaluated previously in Uppsala and Örebro, Sweden, as well as Hamburg, Germany and Alicante, Spain, in comparison with both the broth microdilution reference standard as well as routinely used AST methods such as disc diffusion and MicroScan WalkAway plus, and shown to provide good accuracy and reproducibility for a large selection of bacteria commonly encountered in blood cultures [17–19]. The previous studies have not comprehensively evaluated the reduction in time to result (TTR) and turnaround time (TAT) in comparison to commonly used automated AST methods, however. The aim of the current study was to further investigate how much faster the QuickMIC system would deliver an actionable result in real-life clinical laboratory settings, in this case for laboratories located in Europe and USA. The study focuses on examining the performance a would-be user of QuickMIC rapid AST can expect in a range of real-world clinical settings, including direct comparison with routinely used automated AST systems and comparing the TAT under actual operational conditions. Our hypothesis is that the QuickMIC system can enable same-shift TATs while providing AST results which agree well with the most common routinely used automated AST methods.

## Materials and methods

### Ethics approval

This study was performed on left-over sample material, did not collect human tissue, was non-invasive, non-interventional and did not involve handling of sensitive personal information. Nevertheless, IRB review of the research protocol was initiated at each participating site. The IRB at the Medical College of Wisconsin considered the study IDE exempt (MCW/FH Institutional Review Board #5, ref: PRO00045736) and approved the protocol with waiver of informed consent. The Dr. Balmis University General Hospital IRB approved the research protocol with waiver of informed consent (Comité Ético De Investigación Con Medicamentos Del Departamento Hospital General Universitario De Alicante, ref: CEIm: 2022-086). At the East-Tallinn Central Hospital and the University of North Carolina School of Medicine, IRB review was not deemed necessary by the local IRB based on initial assessment of the project plan, or were covered by existing IRB approval (Research Ethics Committee of the National lnstitute for Health Development (NIHD REC), ref: 2/2022 and Office of Human Research Ethics (OHRE), ref: 19–1149).

### Study design

An observational multicentre study was conducted to evaluate the performance of QuickMIC^®^ GN and the clinical relevance of an ultra-rapid AST system in the clinical diagnostics workflow of sepsis. Leftover blood samples of Gram-negative origin were enrolled in the study between October 2022 to December 2023 at two sites in Europe and two sites in the US (Table 1).

**Table 1:** Details of the study locations

Location	East-Tallinn Central Hospital (EU)	General University Hospital Dr. Balmis (EU)	The Medical College of Wisconsin Inc. (USA)	UNC Medical Center (USA)
Size (beds)	Small (580)	Medium (750)	Large (> 1000)	Large (> 1000)
Blood culture sets/year	6 000	19 000	35 000 – 45 000	35 000 – 40 000
Intensive care units	2	1	10	8
Antibiotic Stewardship Team	Yes	Yes	Yes	Yes
Opening hours of the routine laboratory	08.00–16.00 Mon-Sat	24/7 Daily	24/7 Daily	24/7 Daily
AST services provided	08.00–16.00	08.00–20.00	07.00–18.00	06.30–15.30
Blood culture system	BD BACTEC™ (BD)	BD BACTEC™ (BD)	VersaTREK™ (ThermoFisher)	BD BACTEC™ (BD)
Bacterial ID method	MALDI-ToF (Bruker)	MALDI-ToF (Bruker)	MALDI-ToF (Bruker)	VITEK MS (bioMérieux)
Routine AST system	VITEK^®^2 (bioMérieux)	MicroScan WalkAway plus (BD)	BD Phoenix^™^ (BD)	BD Phoenix^™^ (BD)
AST inoculation method	Rapid pre-incubation (4 h)	Direct-from-PBC	Overnight subculture	Overnight subculture

### Clinical diagnostic workflow

**Fig. 2:**
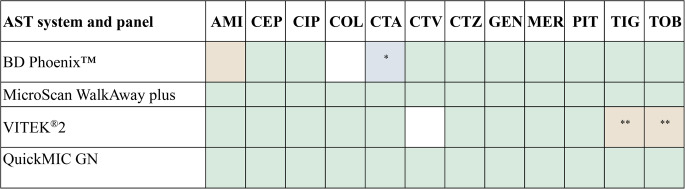
The agreement of antibiotics included in QuickMIC GN and in panels used in the legacy methods. * Cefotaxime (CTA) compared to ceftriaxone, CA comparison only. ** Only one of the two panels used in the laboratory included these agents. Antibiotics included in antibiotic panels used in the current study (green), ceftriaxone used as cefotaxime-equivalent (blue), antibiotics covered in a fraction of the samples included (brown). White indicates antibiotics not included in the antibiotic panel. AMI, Amikacin; CEP, Cefepime; CIP, Ciprofloxacin; COL, Colistin; CTA, Cefotaxime; CTV, Ceftazidime-Avibactam; CTZ, Ceftazidime; GEN, Gentamicin; MER, Meropenem; PIT, Piperacillin-Tazobactam; TIG, Tigecycline; TOB, Tobramycin

Gram staining was executed after blood culture positivity and species identification was performed using MALDI-TOF-MS (Bruker, Germany) at all sites, except UNC Medical Center where VITEK-MS (bioMérieux) was used. At each site, commonly used AST systems were operated largely according to the Manufacturer’s instructions: BD Phoenix™ (BD Biosciences), MicroScan WalkAway plus (Beckman Coulter) or VITEK^®^ 2 (bioMérieux). For MicroScan and VITEK^®^2, the laboratories used direct-from-PBC or inoculation from rapid pre-incubation modifications to the manufacturer’s instructions. The clinical samples were analysed using the QuickMIC system in parallel as it would have been integrated into the diagnostic workflow, and run as a complement to the routine AST method at each site. All sites used QuickMIC^®^ GN (art number: 43 − 001) and the antibiotic panels used by each AST system were as follows: BD Phoenix™ (NMIC 306); MicroScan WalkAway plus (Neg MDR Combo 100, C80306); VITEK^®^ 2 (AST-N330/AST-N332). The antibiotics covered by each panel is summarised in Fig. 2. All antibiotics in the QuickMIC^®^ GN panel were found in the comparator panels, with a few exceptions. Cefotaxime (CTA) was absent from the BD Phoenix™ panel and Ceftriaxone (CTR) was used interchangeably for categorical agreement calculations. Although, Tigecycline (TIG) and Tobramycin (TOB) were included in the VITEK^®^2 system, they were not tested against all samples included in the study (different panels were used).

### Data analysis

If available, the following parameters were recorded: start of blood culture incubation, time of blood culture positivity, and time of Gram-staining. The reported analysis time of each AST system was used as the time to results (TTR). Turnaround time (TAT) was calculated from time of blood culture positivity or time of Gram-staining until time to result (See Table S1 for details). In cases where it had not been practically possible to follow the study protocol, e,g. temporary lack of trained technicians or similar leading to an artificial delay in the start of the QuickMIC instrument of more than 24 h, the samples were excluded from the TAT calculations but included for the comparative performance analysis. Essential Agreement (EA), Categorical Agreement (CA), bias, minor error, major error and very major error were calculated according to performance parameters defined in [20–22]. EUCAST clinical breakpoints v. 15 was applied to determine the antibiotic susceptibility [23]. Multi-drug resistance (MDR) was defined as resistant against at least three antibiotic classes: Aminoglycosides (Amikacin, Gentamycin, Tobramycin), Cephalosporins (Cefepime, Ceftazidime, Cefotaxime), Carbapenems (Meropenem), Polymyxins (Colistin), Glycylcycline (Tigecycline) and Quinolones (Ciprofloxacin).

## Results

### Sample characteristics

In total, 238 unique-patient clinical left-over blood cultures were enrolled in the study and 155 were included in the final data analysis (See Table S2 for details). A total of 19 different bacterial species or genera were collected (See S3 for details) of which ten species or complexes or genera were included in the data analysis (Table 2). The blood cultures were grouped based on the routine AST systems used. Of the total number of blood cultures included, 35.6% and 17.2% were resistant against at least one antibiotic in the BD Phoenix™ and VITEK^®^ 2 data set, while the rate of MDR was 8.2% and 6.9%, respectively. The resistance rate was higher in the MicroScan WalkAway plus data set (47.1%) and the MDR rate was 5.9% (Table 3).

**Table 2:** Distribution of species analyzed by QuickMIC^®^ GN and each routine AST system separately

	BD Phoenix™	MicroScan WalkAway plus	VITEK^®^ 2
*Citrobacter spp.*^*1*^	2	2	0
*Enterobacter cloacae complex* ^*2*^	4	5	1
*Escherichia coli*	40	30	22
*Klebsiella spp.* ^*3*^	22	9	1
*Proteus spp.* ^*4*^	3	1	4
*Pseudomonas aeruginosa*	1	2	0
*Salmonella spp.*	0	1	0
*Serratia spp.*^*5*^	1	2	0
*Stenotrophomonas maltophilia*	1	0	0
*Raultella ornithinolytica*	0	0	1
Total	74	52	29

^1^Includes *Citrobacter koseri*,* Citrobacter freundii*,* Citrobacter braaki*

^2^ Includes *Enterobacter cloacae*,* Enterobacter kobei*,* Enterobacter hormaechei*,* Enterobacter roggenkampii*

^3^Includes *Klebsiella pneumoniae*, *Klebsiella oxytoca and Klebsiella aerogenes*

^4^Includes *Proteus mirabilis* and one unknown species

^5^Includes *Serratia marcescens and Serratia rubidaea*

**Table 3:** Performance characteristics of QuickMIC^®^ GN in comparison to each routine AST system and included resistance phenotypes

	BD Phoenix™	MicroScan WalkAway plus	VITEK^®^2
Essential Agreement (n/n_tot_)	95.9% (659/687)	95.7% (540/564)	96.1 (224/233)
Categorical Agreement (n/n_tot_)	95.8% (597/623)	95.7% (528/552)	96.6 (224/232)
Minor Errors (%)	13 (2.1)	9 (1.6)	2 (0.9)
Major Errors (%)	2 (0.3)	1 (0.2)	2 (0.9)
Very Major Errors (%)	11 (1.8)	14 (2.5)	4 (1.7)
Overall Bias	−10.67	−4.19	+ 7.43
Number of susceptible (%)	549 (88.1)	486 (88.0)	215 (92.7)
Number of intermediate (%)	7 (1.1)	9 (1.6)	1 (0.4)
Number of resistant (%)	67 (10.8)	57 (10.3)	16 (6.9)
MDR rate*	8.2	5.9	6.9
*Percentage of isolates with MDR phenotype, defined as R to at least 3 different antibiotic classes			

Essential Agreement is defined as the percentage of MIC values ± 1 twofold dilution from comparator. Categorical Agreement is the percentage of interpreted categories (Susceptible, intermediate or resistant) aligning with the comparator. The percentage of errors are defined as follows: Minor error, samples that were intermediate by one method and resistant or susceptible by the other; Major error, samples that were resistant by QuickMIC^®^ GN and susceptible by the routine method; Very Major Errors, samples that were susceptible by QuickMIC^®^ GN and resistant by the routine method. Bias was calculated as per ISO 20776-2:2021 and the acceptable range is ± 30%. The EUCAST Breakpoint version v. 15 was used for susceptibility testing. Susceptible: sensitive against all antibiotics included; Intermediate and Resistant: intermediate and resistant against at least one antibiotic included respectively; MDR: multi-drug resistant i.e. resistant against at least three antibiotic classes

### Comparative performance

**Fig. 3:**
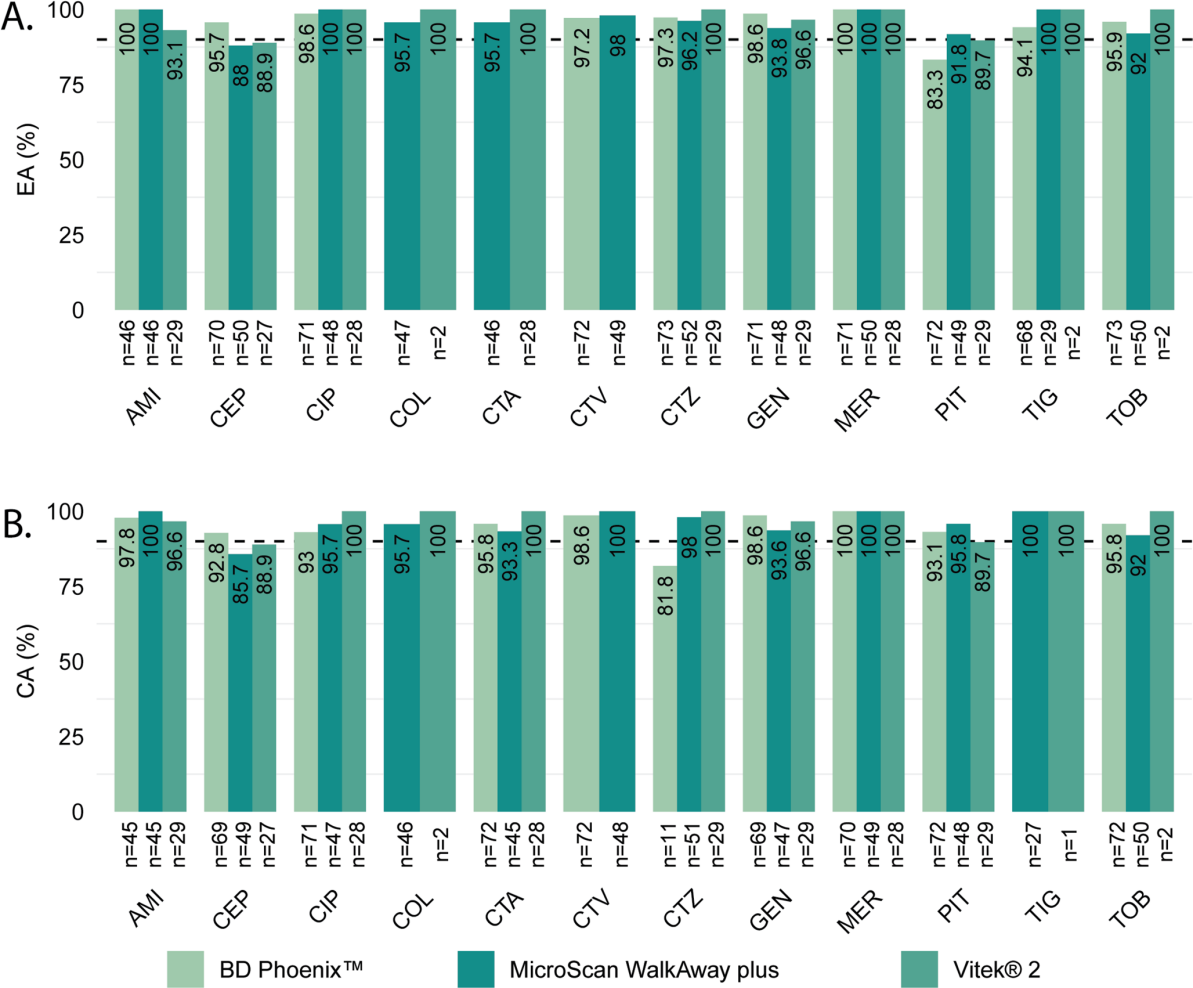
Categorical agreement (**A**) and essential agreement (**B**) between QuickMIC^®^ GN and each legacy method separately. The size of the dataset is indicated by “n” and the dashed line indicates the performance criteria of ≥ 90%. AMI, Amikacin; CEP, Cefepime; CIP, Ciprofloxacin; COL, Colistin; CTA, Cefotaxime; CTV, Ceftazidime-Avibactam; CTZ, Ceftazidime; GEN, Gentamicin; MER, Meropenem; PIT, Piperacillin-Tazobactam; TIG, Tigecycline; TOB, Tobramycin

The performance characteristics of the QuickMIC^®^ GN were determined in comparison to each routine AST system individually (Table 3). The overall EA and CA between QuickMIC^®^ GN and each routine AST system was > 95% (EA and CA 95.7–96.1% and 95.7–96.6%, respectively) while the overall bias as compared to each method was within the acceptable range of ± 30%. Essential and categorical agreement was also determined for each antibiotic and was acceptable, either close to or above the 90% threshold (Fig. 3). For no antibiotic the performance was systematically poor when compared to all three automated systems. For a detailed breakdown of the QuickMIC results vs. the comparative systems, see Figure S5 and Tables S6–S8.

### Time to result

**Fig. 4:**
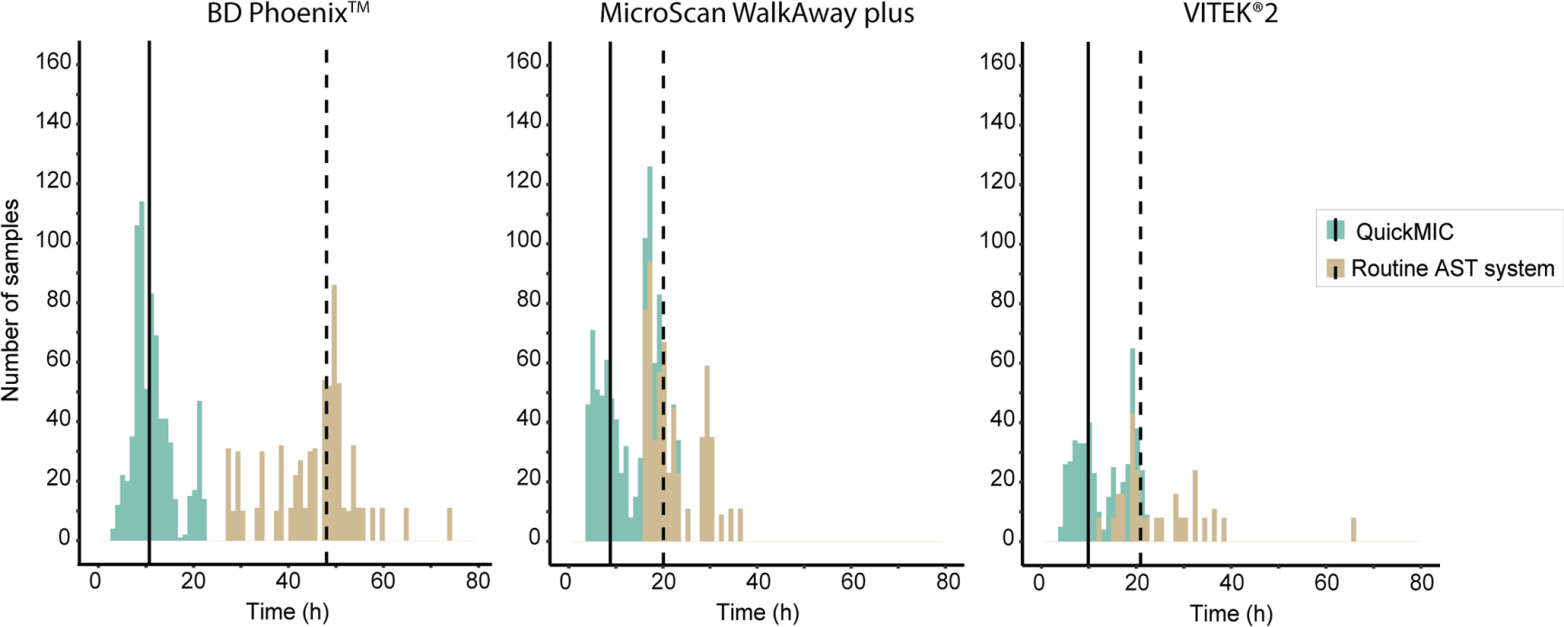
Turnaround time (TAT) of the QuickMIC system in comparison to routine AST systems. Turnaround time is defined as gram-staining or blood culture positivity until a reportable MIC value is available. The solid and dashed line indicates the mean for QuickMIC and the comparison method, respectively

The time to result, i.e. the time from start of each AST analysis until availability of a reportable MIC values as provided in the test report was compared between the systems. The only exception was the MicroScan WalkAway plus system which did not store the analysis time. For this reason, a template time of 16 h was applied, based on previously published research [5, 24]. The average time to result of the QuickMIC system across all three datasets was 3 h and 4 min, while the routine automated AST systems required approximately from 9 to 19 h for completion (Table 4). For detailed per-antibiotic TTR for QuickMIC, please see Table S3. The turnaround time (TAT) was calculated as the time from blood culture positivity or Gram staining until a reportable MIC value was available. Cases where the procedure deviated significantly from the study protocol were excluded, such as when samples were run on the QuickMIC system with a >24 h delay from PBC positivity or Gram-staining. Overall, the QuickMIC system mean TAT ranged from 10 h 18 min to 11 h 30 min. This highlights an important advantage of rapid AST systems, as the TAT was significantly reduced in comparison to each routine AST system; VITEK^®^2, 25 h 18 min; MicroScan WalkAway plus, 22 h 6 min and BD Phoenix™, 45 h 24 min (Table 4; Fig. 4), representing a decrease of 14 h, 12 h and 33 h from clinical routine, respectively. Most of the laboratories were open 24/7, except for one laboratory (Table 1). For this laboratory, 82% of QuickMIC results (282/342) were available within the opening hours the same day the PBC turned positive vs. 0% (0/238) for the comparator method (VITEK^®^2). While the 24/7 laboratories could potentially handle results any time, they do not provide AST services and reporting around the clock, however. For these settings, 83% (492/593, Alicante) and 64% (188/293, North Carolina) − 89.5% (401/448, Wisconsin) of all samples were available during AST service hours the same day where the PBC blood culture turned positive, but none of the comparator method samples were.

**Table 4:** Time to result (TTR) and turnaround time (TAT) for each dataset

	TTR (h ± sd, Q1-Q3)		TAT (h ± sd, Q1-Q3)	
	Automated AST	QuickMIC	Automated AST	QuickMIC
BD Phoenix™	17:33 ± 02:58, 15:32–19:13	03:05 ± 00:26, 02:50–03:20	45:24 ± 09:24, 08:33–13:33	11:30 ± 04:24, 08:33–13:33
MicroScan WalkAway plus	16^1^	03:04 ± 00:28, 02:40–03:20	22:06 ± 05:30, 16:56–28:01	10:18 ± 05:00, 06:02–14:39
VITEK^®^2	09:59 ± 01:46, 08:55–10:26	03:04 ± 00:26, 02:50–03:20	25:18 ± 10:18, 19:14–30:38	11:30 ± 05:12, 07:29–15:23
Overall	**NA**^**2**^	**03:05 ± 00:27**,** 02:50–03:20**	**NA**^**2**^	**11:06 ± 04:48**,** 07:43–14:23**

^1^A template time of 16 h was obtained from PMID: 35,453,179 and PMID: 39,545,740 for MicroScan WalkAway plus as the system did not store the analysis time

^2^ Averages not applicable due to methodological differences between the methods and their implementation at each laboratory

## Discussion

**Fig. 5:**
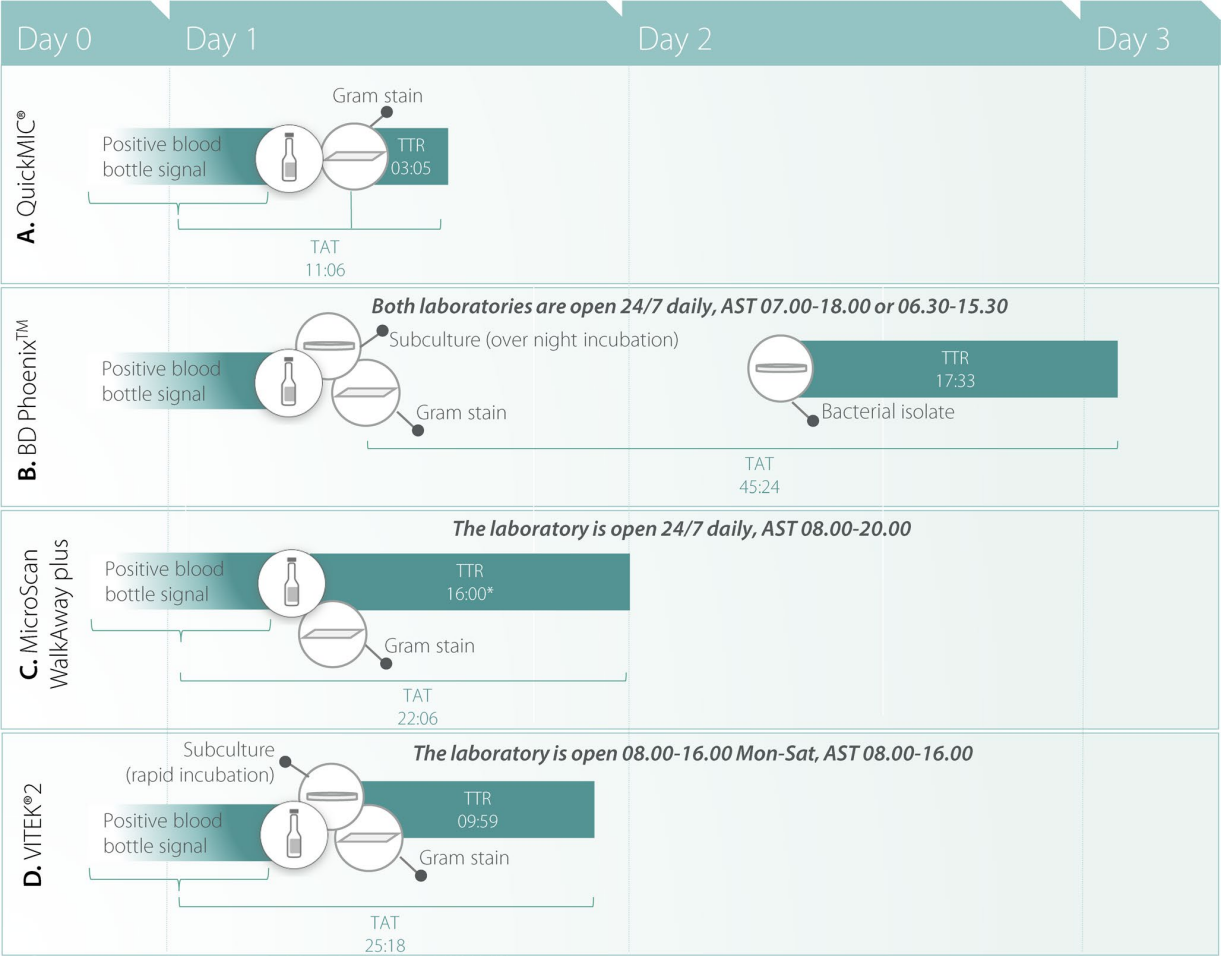
General workflow, average time-to-results (TTR) and average turnaround time (TAT) of included AST systems. Calculations of TTR and TAT were made for each routine AST system separately and they were handled as three separate datasets. In **A**, the TTR and TAT shown for QuickMIC is the average time across all three routine data sets. **B**. Two clinical data sets based on the BD Phoenix^™^ system have been combined. AST was performed between 07–18 or 06.30-15.30 depending on site, and positive blood culture bottles were cultured for overnight incubation as soon as they turned positive. **C**. The site handled positive blood cultures as soon as they turned positive during 08.00–20.00 and performed AST directly from diluted PBC according to an in-house protocol. **D**. During opening hours of the laboratory, positive blood cultures were handled as soon as they turned positive. Rapid incubation according to an in-house protocol was initiated before 11 am, alternatively incubated overnight for AST the following day.––

The purpose of the current study was to evaluate the performance of the QuickMIC system as a tool for guiding antibiotic therapy in patients with bacterial sepsis and bloodstream infection in a clinical setting, as compared to the most commonly used automated AST systems and with real-world routine workflows. In recent years, there has been a major effort to develop rapid AST technologies to decrease the time until guided antibiotic therapy. Time to appropriate treatment is a crucial factor in treatment of sepsis and septic shock, as early appropriate treatment is closely linked with mortality [3, 4]. Overall through the four clinical settings in this study the average TTR for the QuickMIC instrument was 3 h and 4 min (± 28 min) (Table 4), which was on average 7 to 15 h faster than the routine automated AST systems in use at each site. In general, AST systems commonly used in clinical routine in Europe and the US reports an actionable Minimal Inhibitory Concentration (MIC) value within 4–18 h, and traditional phenotypic methods generally need >20 h [5]. While the average TTR of QuickMIC was ~ 3 h, it is important to highlight that the QuickMIC TAT in the current study was on average ~ 11 h, which reflects the large impact of opening hours and laboratory workflows on the TAT. Since positive blood cultures can signal anytime during the day, the availability of tests and the opening hours of the laboratory determines when AST can be started. Even though the majority of participating laboratories were open 24/7, AST testing was only handled during daytime and evenings but not night-time. The protocols in use also have a large impact on TAT, as for example in Tallinn routine AST testing was started from 4 h short-incubation plates, while in Alicante the laboratory uses an in-house direct-from-PBC method for inoculation of the MicroScan Walkaway plus system. In both US laboratories routine AST was on the other hand started from isolated colonies from overnight subculture plates. These protocol differences result in large TAT differences between the routine systems, even though their individual TTR times are similar (Fig. 5). As such, while short TTRs can enable short TATs, the actual performance of rapid AST methods is highly dependent on the implementation in the laboratory workflow. Still, these results demonstrate that a relatively small reduction in TTR (~ 3 vs. 10–16 h, i.e. 7–13 h less) can have a great impact on TAT (10–12 h vs. 22–45 h, i.e. 12–33 h less). This is important because a very short TTR may enable same-shift reporting of results for the samples started earlier during the shift, whereas a longer TTR will likely mean result read-out and reporting happens the next day anyway. As an example from the locations in this study, only the QuickMIC system generated results which were available for initiating clinical action when AST services and staff were available for same-day reporting.

As mentioned, several rapid AST systems have been introduced to clinical use in recent years, but their ability to deliver a rapid AST result is similarly impacted by their TTR in comparison to the opening hours of the laboratory and the possibility of the clinical staff to act on the rapid AST result and provide a treatment intervention. The current study shows that the QuickMIC system could facilitate rapid management of patients with bloodstream infection and sepsis despite factors such as limited opening hours, due to the short TTR. Previously investigated commercially available rapid AST systems in contrast have average TTR of 5–7 h [25–27] which presents problems in the context of same-shift AST [7]. Since most results would not be available to the clinical team to be acted upon until the next day, the impact of these rapid AST technologies is reduced in comparison to the standard, routinely used automated AST systems. Even if there are an increasing amount of 24/7 laboratories available, the staff capacity is usually not the same during the night, which means that advanced diagnostics like rapid AST can be unavailable during these working shifts, as was the case in the current study. Furthermore, even if the laboratory can deliver an actionable AST result or MIC-value 24/7, the availability of senior physicians or stewardship teams is likely to be reduced off-hours, which prevents clinically important actions to be taken based on the result. Therefore, adjustments to current diagnostic workflows might be required for optimal clinical implementation of rapid AST technologies, but the short TTR provided by the QuickMIC system can reduce the magnitude of adjustments required, since 2–4 h TTR means same-shift AST reporting is possible for samples arriving throughout most of the working day, as demonstrated here. Furthermore, it is important to consider that shorter AST TTRs also means supporting tests such as organism identification (ID) must be optimized to maximize the benefit of the rapid AST methods. Traditional culture-based ID methods are too slow to enable categorisation of the AST result, and many laboratories have yet to adopt rapid ID methods such as direct-from-PBC syndromic panels or short-incubation MALDI-TOF protocols. In this study, all laboratories had the capacity to provide a rapid ID before or shortly after the QuickMIC result, but that might not always be the case. Again, this emphasizes the need to consider the entire diagnostic workflow to enable rapid result TATs to enable earlier guided antibiotic therapy, and not focus on individual methods only.

While rapid AST is widely seen as necessary to improve the management of bacterial infections, especially in the age of antibiotic resistance where empirical therapy is increasingly inadequate, new and advanced technologies such as rapid AST systems come with a higher per-sample cost than traditionally used AST methods. This increased cost of testing must be set in relation to the overall potential benefit on reduced mortality, length-of-stay, and improved stewardship measures such as empirical broad-spectrum antibiotic use, which has been variously reported in prior studies [28–31]. The increased per-sample costs associated with new rapid AST systems can potentially be mitigated by method development and improvement of traditional low-cost AST methods, the EUCAST RAST protocol for rapid read-out of standard disc-diffusion is one such attempt [8]. Improved, rapid traditional protocols are often also associated with increased costs and/or complexity however, for example due to more complicated handling and personnel requirements which may require expensive automation for adoption [12]. Improved traditional low-cost methods may furthermore have significant downsides. As an example, even if a phenotypic AST result can be generated by the EUCAST RAST protocol as early as after 4 h of incubation on a plate, there are several limitations for certain drug-bug combinations [8–11], and the ATU (area of technical uncertainty) can be very wide at the early timepoints. Also, the RAST protocol can only generate categorical interpretation (and not a MIC value) which further limits the use for guiding therapy, especially in highly resistant environments where treatment with antibiotic combinations or higher dosages may be attempted for MDR isolates with “close to the breakpoint” MIC results. The manual interpretation of growth zones requires experienced staff which may also not always be available, for example during nighttime. An alternative which may mitigate some of these issues are various efforts for off-label or LDT adaptations for inoculating AST instruments directly with PBC broth, as reported elsewhere [32–36], and also as used by one laboratory in this study. While these studies demonstrate encouraging results with high accuracy and a large time-gain in multiple AST systems, complex manual protocol modifications may be necessary to reduce human cell contamination when inoculating direct-from-PBC. There are studies that indicate that direct-from-PBC inoculation can produce results in high agreement with the standard overnight isolate protocol also without complicated sample preparation, however [34]. The main obstacle for these adaptations is that off-label use and LDT modifications of commercial systems is difficult and time-consuming, and must be performed on a per-lab basis until the manufacturer can certify direct PBC inoculation as a supported on-label method with the regulatory authorities. LDT modifications are also highly discouraged in some legislations, such as under the new in-vitro device regulation (IVDR) in the EU, where LDT and off label use is only allowed if no commercial alternatives exist; and also is associated with significant regulatory overhead for the laboratory [37, 38].

This study has some important limitations. Firstly, this study was not designed to measure the regulatory performance of the QuickMIC system, i.e. the accuracy, precision, bias and reproducibility as compared to the reference standard method broth microdilution (for that we refer to the previous publication of the EU regulatory performance data [18]), but instead to investigate how the system performs compared to the methods and workflows in actual use, with emphasis on TAT. This is a significant limitation of the performance data presented here, and the accuracy data as compared to each system should only be taken as an indicator that QuickMIC can produce results which agree well with the comparator legacy automated methods in clinical conditions. The exact performance figures are likely to differ due to the small sample size, poor representation of important species such as *K. pneumonia* and *P. aeruginosa* encountered at each site, and the biases inherent in comparing to multiple different methods. While this means care should be taken when generalizing the accuracy and bias results presented here, the main purpose of this study was to investigate the effect on improved TAT by using a system with a very short TTR, under a variety of workflows and laboratory settings. Secondly, another limitation of the present study is the lack of data from PBC broth from the BactAlert Virtuo (bioMérieux) blood culture system, which has been demonstrated to be more sensitive and generate PBCs with a lower TTR than competing systems. This would not impact the TAT results demonstrated here, since they were measured starting from blood culture positivity until actionable result. However, a shorter blood-culture TTR can result in a positive blood culture with lower bacterial loads, which potentially could affect direct-from-PBC AST performance. The previously mentioned regulatory study did include the Virtuo system however, and did not demonstrate any significant dependency of the inoculate concentration on the QuickMIC result [18]. For the current study, 10 samples corresponding to 115 datapoints (ca. 13% of the dataset) were run within 2 h of positivity, which can result in low inoculum on the included blood culture systems.

In summary, the QuickMIC ultra-rapid AST system shows good performance with an average essential and categorical agreement of 95.7–96.1% and 95.7–96.6% respectively, across all sites and comparative routinely used automated AST systems included in the current study, while on average reducing the TAT with 53–75% and enabling same-shift AST results in laboratories with restricted service hours. This has the potential to improve the management of patients with bacterial infections, especially in clinical settings where the antibiotic resistance is high and there is a high rate of inadequate empirical therapy; which could include e.g. geographic areas such as southern Europe, but also specific patient populations such as individuals wounded in conflict areas with high incidence of MDR infections but who are treated elsewhere. There are significant limitations with the QuickMIC system in its currently investigated configuration, however. Mainly, the QuickMIC system supports fewer antibiotics (12 cleared for use in the EU under the CE label) compared to the routinely used comparator methods, as well as a limited panel of supported species. The selection of antibiotics available on the panel examined in this study may also be less useful for de-escalation purposes. Furthermore, the cost-per-test is significantly higher compared to the currently used routine methods. It remains to be investigated whether these limitations are worth the potential added benefit of reduced TTR and TAT, especially in the target population of patients with bloodstream infection and/or sepsis.

## Supplementary Information

Below is the link to the electronic supplementary material.

ESM 1 (DOCX 299 KB)

## Data Availability

All data is available upon reasonable request to the corresponding author.
