# Supplementary material for: Multicenter evaluation of the QuickMIC® rapid AST system in clinical practice: impact on turnaround time compared to routine AST systems
Source: Eur J Clin Microbiol Infect Dis. 2025 Oct 13;45(1):185–96. doi: 10.1007/s10096-025-05298-z (PMC12872699; doi:10.1007/s10096-025-05298-z)
Supplement: Supplementary file 1 — (DOCX 299 KB) [file 10096_2025_5298_MOESM1_ESM.docx]

Supplementary material for: QuickMIC rapid AST in clinical routine – evaluation of accuracy and speed in comparison to routinely used automated AST systems

Anna Olsson^1^, Alexandra Rafeletou^1^, Amanda Åman^1^, Nicolette Athanasiou^2^, Celia García-Rivera^3^, Derek Gerstbrein^2^, Brian Mesich^2^, Logan Patterson^4^, Julia Vahturova^5^, Nicole VanZeeland^2^, Andrea Ricart Silvestre^3^, Kevin Alby^6^, Matthew L. Faron^2^, Jessica Hoff^2^, Marina Ivanova^5^, Juan Carlos Rodríguez Díaz^3^, Cecilia Johansson^1^, Christer Malmberg^#, 1,6^

^1^Gradientech AB, Uppsala, Sweden.

^2^Department of Pathology, Medical College of Wisconsin, Milwaukee, WI, USA.

^3^Dr. Balmis University General Hospital, Alicante Institute for Health and Biomedical Research (ISABIAL), Alicante, Spain

^4^Department of Pathology and Laboratory Medicine, Medical University of South Carolina, Charleston, SC, USA.
^5^Clinical Microbiology Laboratory, East-Tallinn Central Hospital, Tallin, Estonia

^6^Department of Pathology and Laboratory Medicine, University of North Carolina School of Medicine, Chapel Hill, NC, USA.

^7^Department of Medical Sciences, Uppsala University, Uppsala, Sweden.

# Corresponding author

Christer Malmberg

Postal adress: Gradientech AB, Dag Hammarskjölds väg 36B, 752 37 Uppsala

Email address: [christer.malmberg@gradientech.se](mailto:christer.malmberg@gradientech.se)

ORCID: <https://orcid.org/0000-0003-3187-8732>

Table S1. Details on turnaround time calculations for each data set

| **Site** | **TAT start** | **TAT end** |
| --- | --- | --- |
| VITEK^®^ 2 | Blood culture positivity | Reportable MIC value available |
| MicroScan WalkAway plus | Blood culture positivity | 16 hours after start^1^ |
| BD Phoenix™ | Gram staining^2^ | Reportable MIC value available |

^1^A template time of 16 hours was applied from PMID: 39194288, as the instrument did not store this information. ^2^ Time of Gram-staining was used instead of blood culture positivity, as this information was not available in the laboratory LIS.

Table S2. Overview of the number of left-over blood cultures included in the study

|  | **East-Tallinn Central Hospital** | **General University Hospital Dr. Balmis** | **The Medical College of Wisconsin Inc.** | **UNC Medical Centre** | **Total** |
| --- | --- | --- | --- | --- | --- |
| Enrolled left-over blood cultures | 37 | 90 | 59 | 52 | 238 |
| Samples lacking legacy AST data | 4 | 17 | 7 | 18 | 46 |
| Polymicrobial, anaerobic, fastidious or gram-positive samples | 2 | 1 | 0 | 0 | 3 |
| Infection other than bloodstream | 0 | 0 | 0 | 4 | 4 |
| Other (instrument or user error) | 2 | 20 | 6 | 2 | 30 |
| **Included in the final dataset** | **29** | **52** | **46** | **28** | **155** |

Table S3. Number of species included in the study at each study site

|  | **East-Tallinn Central Hospital** | **General University Hospital Dr. Balmis** | **The Medical College of Wisconsin Inc.** | **UNC Medical Centre** |
| --- | --- | --- | --- | --- |
| *E. coli* | 22 | 30 | 21 | 19 |
| *K. pneumoniae* | 1 | 8 | 11 | 8 |
| *K. oxytoca* | 0 | 0 | 2 | 0 |
| *K. aerogenes* | 0 | 1 | 1 | 0 |
| *C. braaki* | 0 | 1 | 0 | 0 |
| *C. koseri* | 0 | 0 | 1 | 0 |
| *C. freundii* | 0 | 1 | 1 | 0 |
| *P. mirabilis* | 4 | - | 2 | 1 |
| *Proteus spp.* | - | 1 | - | - |
| *P. aeruginosa* | 0 | 2 | 1 | 0 |
| *S. marcescens* | 0 | 1 | 1 | 0 |
| *S. rubidae* | 0 | 1 | 0 | 0 |
| *S. maltophilia* | 0 | 0 | 1 | 0 |
| *E. cloacae* | 1 | 1 | 4 | 0 |
| *E. kobei* | 0 | 1 | 0 | 0 |
| *E. hormaechei* | 0 | 2 | 0 | 0 |
| *E. roggenkampii* | 0 | 1 | 0 | 0 |
| *Salmonella* spp. | 0 | 1 | 0 | 0 |
| *R. ornithinolytica* | 1 | 0 | 0 | 0 |
| **Total** | **29** | **52** | **46** | **28** |

*Table S4 – QuickMIC TTR per antibiotic*

| **Antibiotic** | **TTR** | **SD** | **Q1** | **Q3** |
| --- | --- | --- | --- | --- |
| AMI | 02:27 | 00:14 | 02:20 | 02:30 |
| CEP | 03:11 | 00:19 | 03:00 | 03:20 |
| CIP | 03:13 | 00:28 | 02:50 | 03:48 |
| COL | 02:52 | 00:25 | 02:40 | 03:00 |
| CTA | 03:27 | 00:18 | 03:20 | 03:50 |
| CTV | 02:55 | 00:18 | 02:50 | 03:00 |
| CTZ | 03:18 | 00:20 | 03:00 | 03:30 |
| GEN | 03:09 | 00:28 | 02:50 | 03:30 |
| MER | 03:05 | 00:26 | 02:40 | 03:20 |
| PIT | 03:13 | 00:17 | 03:00 | 03:20 |
| TIG | 03:12 | 00:27 | 02:50 | 03:40 |
| TOB | 02:54 | 00:23 | 02:40 | 03:10 |

Figure S5. Bias plots of QuickMIC GN vs. Phoenix, VITEK2 and MicroScan Walkaway


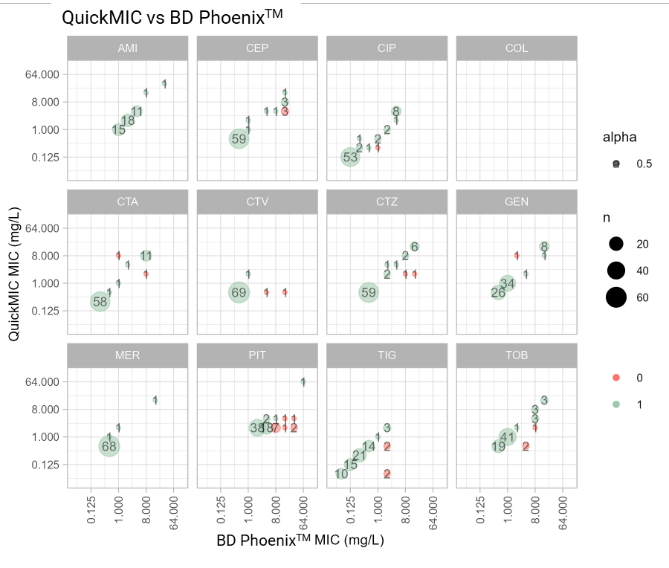

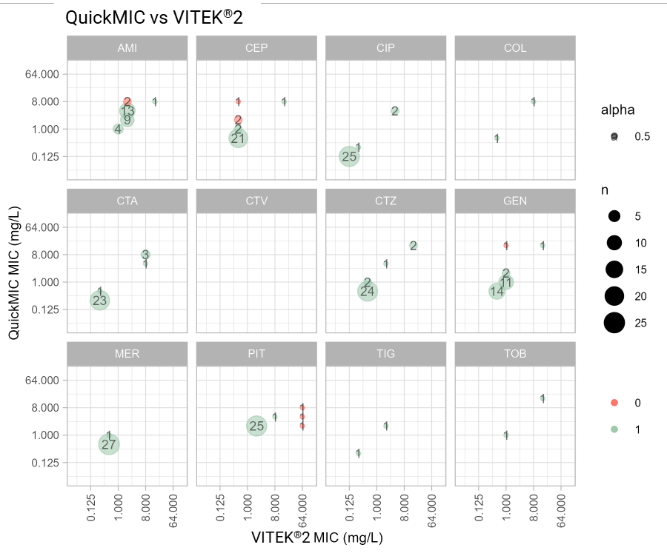


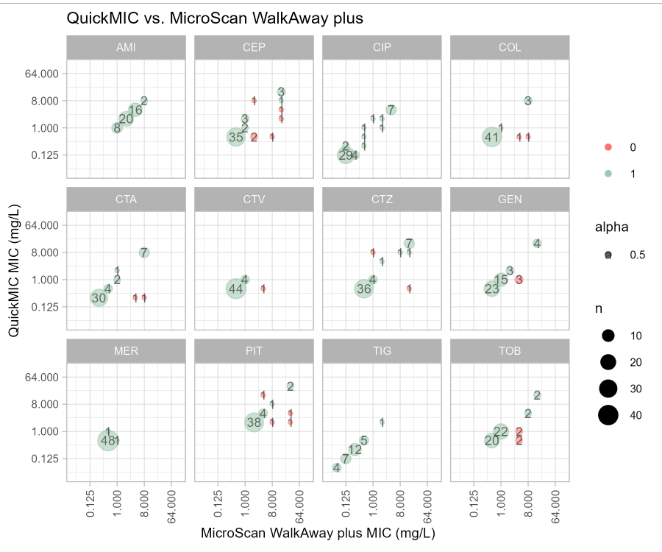


*Figure S5. Comparison of QuickMIC MIC results with the MIC results from the three automated AST systems included in the study. On the y-axis are the QuickMIC results, and the x-axis shows the respective comparator result. The dot size and number indicates the number of strains with the same result, and green color indicates a result where QuickMIC was in Essential Agreement with the comparator method, while a red dot indicates a result not in agreement with the comparator method. It is worth noting that a result may in actuality be an error in either QuickMIC or the comparator method, as compared to what the reference method broth microdilution may have indicated.*

*Table S6 – Detailed data for agreement calculations between BD Phoenix^TM^ and QuickMIC. AB: antibiotic, N: nr of runs with MIC results for both methods, Nc: nr of runs with categorical data for both methods, Ns: nr of strains run on both methods, CA: categorical agreement, EA: essential agreement, S,I,R: nr of susceptible/intermediate/resistant strains according to the comparator method, MiD: minor deviations, MD: major deviations, VMD: very major deviations, OS: nr of strains with on-scale results for both methods.*

| **AB** | **N** | **N_c_** | **N_s_** | **CA** | **EA** | **S** | **I** | **R** | **MiD** | **MD** | **VMD** | **Bias** | **OS** |
| --- | --- | --- | --- | --- | --- | --- | --- | --- | --- | --- | --- | --- | --- |
| AMI | 46 | 45 | 74 | 44 (97.8) | 46 (100) | 45 | 0 | 0 | 0 (0) | 1 (2.2) | 0 (0) | 2.22 | 0 |
| CEP | 70 | 69 | 70 | 64 (92.8) | 67 (95.7) | 61 | 1 | 7 | 5 (7.2) | 0 (0) | 0 (0) | -8.41 | 5 |
| CIP | 71 | 71 | 73 | 66 (93) | 70 (98.6) | 55 | 2 | 14 | 4 (5.6) | 0 (0) | 1 (1.4) | -8.25 | 7 |
| COL | - | - | - | - | - | - | - | - | - | - | - | - | - |
| CTA* | - | 72 | 74 | 69 (95.8) | - | 61 | 1 | 10 | 2 (2.8) | 1 (1.4) | 0 (0) | - | - |
| CTV | 72 | 72 | 73 | 71 (98.6) | 70 (97.2) | 71 | 0 | 1 | 0 (0) | 0 (0) | 1 (1.4) | -65.26 | 16 |
| CTZ | 73 | 11 | 73 | 9 (81.8) | 71 (97.3) | 0 | 2 | 9 | 2 (18.2) | 0 (0) | 0 (0) | -1.22 | 7 |
| GEN | 71 | 69 | 71 | 68 (98.6) | 70 (98.6) | 60 | 0 | 9 | 0 (0) | 0 (0) | 1 (1.4) | -1.20 | 1 |
| MER | 71 | 70 | 74 | 70 (100) | 71 (100) | 70 | 0 | 0 | 0 (0) | 0 (0) | 0 (0) | 2.86 | 1 |
| PIT | 72 | 72 | 73 | 67 (93.1) | 60 (83.3) | 65 | 1 | 6 | 0 (0) | 0 (0) | 5 (6.9) | -88.57 | 33 |
| TIG** | 68 | - | 70 | - | 64 (94.1) | - | - | - | - | - | - | -5.88 | 6 |
| TOB | 73 | 72 | 73 | 69 (95.8) | 70 (95.9) | 61 | 0 | 11 | 0 (0) | 0 (0) | 3 (4.2) | -8.22 | 9 |
| **Overall** | **687** | **623** | **74** | **597 (95.8)** | **659 (95.9)** | **549** | **7** | **67** | **13 (2.1)** | **2 (0.3)** | **11 (1.8)** | **-10.67** | **85** |

* CTA on the QuickMIC panel was compared with ceftriaxone on the Phoenix panel, for CA only. ** The TIG measurement ranges for QuickMIC and Phoenix did not overlap due to differences in breakpoints between CLSI and EUCAST, making categorical comparison impossible.

*Table S7 – Detailed data for agreement calculations between VITEK®2 and QuickMIC.*

| **AB** | **N** | **N_c_** | **N_s_** | **CA** | **EA** | **S** | **I** | **R** | **MiD** | **MD** | **VMD** | **Bias** | **OS** |
| --- | --- | --- | --- | --- | --- | --- | --- | --- | --- | --- | --- | --- | --- |
| AMI | 29 | 29 | 29 | 28 (96.6) | 27 (93.1) | 28 | 0 | 1 | 0 (0) | 0 (0) | 1 (3.4) | 48.28 | 1 |
| CEP | 27 | 27 | 27 | 24 (88.9) | 24 (88.9) | 26 | 0 | 1 | 2 (7.4) | 1 (3.7) | 0 (0) | -80.77 | 2 |
| CIP | 28 | 28 | 28 | 28 (100) | 28 (100) | 26 | 0 | 2 | 0 (0) | 0 (0) | 0 (0) | 0.00 | 0 |
| COL | 2 | 2 | 29 | 2 (100) | 2 (100) | 1 | 0 | 1 | 0 (0) | 0 (0) | 0 (0) | 0.00 | 0 |
| CTA | 28 | 28 | 28 | 28 (100) | 28 (100) | 24 | 0 | 4 | 0 (0) | 0 (0) | 0 (0) | -20.83 | 2 |
| CTV | - | - | - | - | - | - | - | - | - | - | - | - | - |
| CTZ | 29 | 29 | 29 | 29 (100) | 29 (100) | 26 | 1 | 2 | 0 (0) | 0 (0) | 0 (0) | 11.11 | 5 |
| GEN | 29 | 29 | 29 | 28 (96.6) | 28 (96.6) | 28 | 0 | 1 | 0 (0) | 1 (3.4) | 0 (0) | 10.71 | 0 |
| MER | 28 | 28 | 28 | 28 (100) | 28 (100) | 28 | 0 | 0 | 0 (0) | 0 (0) | 0 (0) |  | 0 |
| PIT | 29 | 29 | 29 | 26 (89.7) | 26 (89.7) | 26 | 0 | 3 | 0 (0) | 0 (0) | 3 (10.3) | -13.79 | 1 |
| TIG | 2 | 1 | 29 | 1 (100) | 2 (100) | 1 | 0 | 0 | 0 (0) | 0 (0) | 0 (0) | 0.00 | 1 |
| TOB | 2 | 2 | 28 | 2 (100) | 2 (100) | 1 | 0 | 1 | 0 (0) | 0 (0) | 0 (0) | 0.00 | 0 |
| **Overall** | **233** | **232** | **29** | **224 (96.6)** | **224 (96.1)** | **215** | **1** | **16** | **2 (0.9)** | **2 (0.9)** | **4 (1.7)** | **7.43** | **12** |

*Table S8 – Detailed data for agreement calculations between MicroScan Walkaway plus and QuickMIC*

| **AB** | **N** | **N_c_** | **N_s_** | **CA** | **EA** | **S** | **I** | **R** | **MiD** | **MD** | **VMD** | **Bias** | **OS** |
| --- | --- | --- | --- | --- | --- | --- | --- | --- | --- | --- | --- | --- | --- |
| AMI | 46 | 45 | 47 | 45 (100) | 46 (100) | 45 | 0 | 0 | 0 (0) | 0 (0) | 0 (0) | 0.00 | 0 |
| CEP | 50 | 49 | 50 | 42 (85.7) | 44 (88) | 40 | 2 | 7 | 6 (12.2) | 0 (0) | 1 (2) | -2.91 | 4 |
| CIP | 48 | 47 | 49 | 45 (95.7) | 48 (100) | 32 | 5 | 10 | 2 (4.3) | 0 (0) | 0 (0) | -26.96 | 8 |
| COL | 47 | 46 | 49 | 44 (95.7) | 45 (95.7) | 41 | 0 | 5 | 0 (0) | 0 (0) | 2 (4.3) | -4.26 | 1 |
| CTA | 46 | 45 | 49 | 42 (93.3) | 44 (95.7) | 37 | 0 | 8 | 1 (2.2) | 0 (0) | 2 (4.4) | -1.72 | 2 |
| CTV | 49 | 48 | 50 | 48 (100) | 48 (98) | 48 | 0 | 0 | 0 (0) | 0 (0) | 0 (0) | -2.04 | 1 |
| CTZ | 52 | 51 | 52 | 50 (98) | 50 (96.2) | 40 | 2 | 9 | 0 (0) | 0 (0) | 1 (2) | 0.81 | 4 |
| GEN | 48 | 47 | 51 | 44 (93.6) | 45 (93.8) | 40 | 0 | 7 | 0 (0) | 0 (0) | 3 (6.4) | -6.25 | 3 |
| MER | 50 | 49 | 50 | 49 (100) | 50 (100) | 49 | 0 | 0 | 0 (0) | 0 (0) | 0 (0) | -98.00 | 1 |
| PIT | 49 | 48 | 49 | 46 (95.8) | 45 (91.8) | 45 | 0 | 3 | 0 (0) | 1 (2.1) | 1 (2.1) | -4.08 | 2 |
| TIG | 29 | 27 | 48 | 27 (100) | 29 (100) | 27 | 0 | 0 | 0 (0) | 0 (0) | 0 (0) | 0.00 | 1 |
| TOB | 50 | 50 | 51 | 46 (92) | 46 (92) | 42 | 0 | 8 | 0 (0) | 0 (0) | 4 (8) | -12.00 | 4 |
| **Overall** | **564** | **552** | **52** | **528 (95.7)** | **540 (95.7)** | **486** | **9** | **57** | **9 (1.6)** | **1 (0.2)** | **14 (2.5)** | **-4.19** | **31** |
